# Daphnia as a model organism to probe biological responses to nanomaterials—from individual to population effects via adverse outcome pathways

**DOI:** 10.3389/ftox.2023.1178482

**Published:** 2023-04-14

**Authors:** Katie Reilly, Laura-Jayne A. Ellis, Hossein Hayat Davoudi, Suffeiya Supian, Marcella T. Maia, Gabriela H. Silva, Zhiling Guo, Diego Stéfani T. Martinez, Iseult Lynch

**Affiliations:** ^1^ School of Geography, Earth and Environmental Sciences, University of Birmingham, Birmingham, United Kingdom; ^2^ Brazilian Nanotechnology National Laboratory (LNNano), Brazilian Center for Research in Energy and Materials (CNPEM), Campinas, Brazil

**Keywords:** ecotoxicity, high throughput, microfluidics, nanosafety assessment, standardised testing, nanomaterials (A)

## Abstract

The importance of the cladoceran *Daphnia* as a model organism for ecotoxicity testing has been well-established since the 1980s. *Daphnia* have been increasingly used in standardised testing of chemicals as they are well characterised and show sensitivity to pollutants, making them an essential indicator species for environmental stress. The mapping of the genomes of *D. pulex* in 2012 and *D. magna* in 2017 further consolidated their utility for ecotoxicity testing, including demonstrating the responsiveness of the *Daphnia* genome to environmental stressors. The short lifecycle and parthenogenetic reproduction make *Daphnia* useful for assessment of developmental toxicity and adaption to stress. The emergence of nanomaterials (NMs) and their safety assessment has introduced some challenges to the use of standard toxicity tests which were developed for soluble chemicals. NMs have enormous reactive surface areas resulting in dynamic interactions with dissolved organic carbon, proteins and other biomolecules in their surroundings leading to a myriad of physical, chemical, biological, and macromolecular transformations of the NMs and thus changes in their bioavailability to, and impacts on, daphnids. However, NM safety assessments are also driving innovations in our approaches to toxicity testing, for both chemicals and other emerging contaminants such as microplastics (MPs). These advances include establishing more realistic environmental exposures via medium composition tuning including pre-conditioning by the organisms to provide relevant biomolecules as background, development of microfluidics approaches to mimic environmental flow conditions typical in streams, utilisation of field daphnids cultured in the lab to assess adaption and impacts of pre-exposure to pollution gradients, and of course development of mechanistic insights to connect the first encounter with NMs or MPs to an adverse outcome, via the key events in an adverse outcome pathway. Insights into these developments are presented below to inspire further advances and utilisation of these important organisms as part of an overall environmental risk assessment of NMs and MPs impacts, including in mixture exposure scenarios.

## 1 Introduction

The zooplankton cladoceran *Daphnia* has captivated biologists for centuries because of its importance in aquatic ecosystems, and its flexibility to cope with, and respond to, environmental stressors. *Daphnia* are a well-established and widely used model organism for freshwater toxicity testing as they are well characterised, have a rapid parthenogenetic reproductive cycle and show sensitivity to a range of environmental xenobiotics. *Daphnia* are also a non-sentient species, meaning that their use in toxicity testing is considered acceptable as a strategy for the reduction, replacement and refinement (NC3Rs) of traditional animal testing, making them an optimal model in ecotoxicology (Colbourne et al., 2022; NC3Rs, 2022). A broad set of behavioural and morphological changes can be observed in *Daphnia* when exposed to environmental stimuli, which forms the foundation of the defined and standardised protocols for chemical toxicity testing, such as the OECD 202 (Acute toxicity) and 211 (Reproduction) tests and the EPA testing of chemicals (OECD, 2004; OECD, 2012; Maxwell et al., 2014). Endpoints evaluated encompass responses such as immobilisation and lethality, which are measured in the acute immobilisation tests in the OECD 202 assay. Changes in life history traits during the chronic test (OECD 211) are also measured, including reproductive changes (such as an increase or decrease in the number of neonates per adult daphnid, or a delay between broods), and growth trends. Further to the standard test end points, phenotypic changes can be observed such as additional spines on the helmet, variability in lipid deposits and behavioural changes such as swimming activity (Colbourne et al., 2011; Chevalier et al., 2015; Karatzas et al., 2020; Tkaczyk et al., 2021). Due to the historic use of *Daphnia* for chemical testing, they are also an optimal model organism for testing challenging and emerging toxicants such as nanomaterials (NMs) and microplastics (MPs) (Nasser and Lynch, 2019; Zimmermann et al., 2020).

NMs, as described by the European Commission, have at least one dimension less than 100 nm (European Commission. Joint Research Centre, 2020). NMs exist in the environment from a range of sources, including naturally occurring (e.g., volcanic ash), incidental particles formed as a result of human activities (e.g., combustion particles, secondary MPs) or can be engineered/manufactured by industry at the nanoscale to exploit specific properties (Jeevanandam et al., 2018). During synthesis, NMs are normally coated with ligands to control their size and limit their agglomeration (Buesser and Pratsinis, 2012). They are distinguished from other non-nanoscale materials by their unique physico-chemical properties (Haase and Lynch, 2018). Being small in size, NMs have a larger surface areas per unit mass than bigger particles, which makes them highly reactive and more dynamic in environmental systems, giving them the ability to interact with different molecules and biological systems (Rosenkranz et al., 2009; Markiewicz et al., 2018; Nasser et al., 2020) which can transform their original identity (Lowry et al., 2012; Spurgeon et al., 2020).

MPs are a significant environmental concern due to their ubiquitous presence, increased biological interactions (compared to macroscale plastic) and difficulties in sampling. Although the size classification of microplastic is often discussed within the literature, the most frequently used definition of MPs is the National Oceanic and Atmospheric Administration (NOAA) definition of less than 5 mm (NOAA, 2023), but discussions within the research community are underway to re-evaluate this in line with the advanced analytical methods now being developed and implemented (Hartmann et al., 2019). MP are also often reported by morphology, categorised as beads or spheres, fibres and fibre bundles, pellets, film, foam or fragments and are introduced into the environment as either primary or secondary plastic (Rochman et al., 2019).

In theory, the considerations applied to NMs for ecotoxicity testing can also be applied to MPs, as the physical interactions and surface conditioning of MPs will also occur in their local environments. Although a relatively emerging field, *Daphnia* have already been used for a range of MP toxicity studies to date, to elucidate the potential impacts that MPs induce in freshwater environments (Nasser and Lynch, 2016; Schür et al., 2020; Zimmermann et al., 2020; Kelpsiene et al., 2022).

Nanomaterials and microplastics are challenging toxicants to assess due to their physical nature and the surface area of the particles, which makes them an interesting lens from which to review the development in the field of ecotoxicology. *Daphnia* are a fantastic model for toxicity assessment due to their filter feeding mechanism which means that particle uptake is highly likely, and their transparent bodies then enables a range of optical methods to be applied and developed to quantify the uptake of the physical toxicants, which in turn leads to novel approaches compared to those available for assessment of toxicity of the soluble chemicals that have historically been assessed (Nasser and Lynch, 2019). The particle surface also poses an interesting aspect of the ecotoxicological assessment; the surface of the particles is dynamic and will be affected by the local environment including by biomolecules released by the *Daphnia*, leading to a more changeable and complex relationship between the toxicant and model organism than that for soluble chemicals (Wheeler et al., 2021; Reilly et al., 2022). These dynamic surface-driven properties, which are shared between microplastics and nanomaterials, have enabled interesting developments across the field of ecotoxicology, leading to the development of techniques for assessing *in situ* transformations and eco-corona evolution. Technological advances such as lab on a chip (Section 7) and conceptual frameworks for identification of key (molecular) events that contribute to adverse outcome pathways (Section 6) provide exciting avenues for further research and development (Mortensen et al., 2022).

## 2 Nanomaterial transformations in the environment and the role of *Daphnia*

**FIGURE 1:**
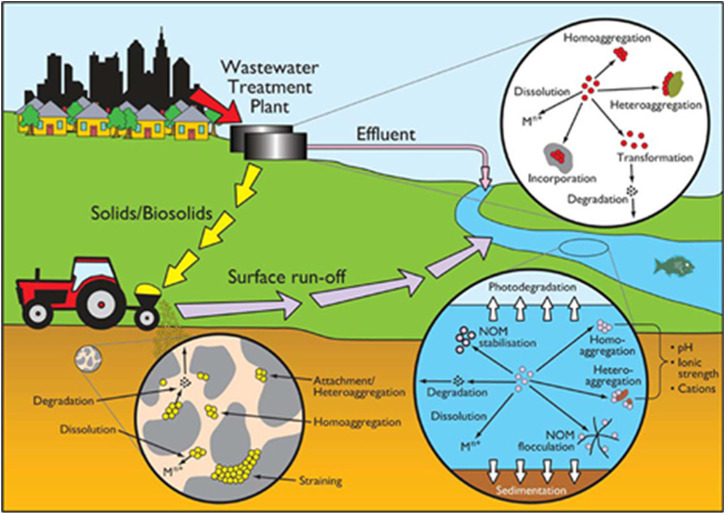
Transformation processes of NMs in the environment. Reproduced from (Batley et al., 2013) with permission from ACS Publications.

When NMs are released into the environment, they interact with many environmental components and go through various dynamic transformation processes which can change their physico-chemical properties (Abbas et al., 2020; Malakar et al., 2021; Wheeler et al., 2021), and significantly impact their toxicity, reactivity, fate and transport in both environmental and biological systems (Ellis and Lynch, 2020; Wheeler et al., 2021; Reilly et al., 2022). Transformation processes such as adsorption of molecules/ions and macromolecules, agglomeration, oxidation/reduction (redox) reactions, sulfidation and dissolution all occur in biological and environmental systems and can greatly affect the behaviour of NMs (Lowry et al., 2012; Spurgeon et al., 2020). The physicochemical transformation of NMs under different environmental conditions are driven by several variables such as ionic strength, kinetics, pH, stability, synthesis method, valency, capping agent, and cation type (Mitrano et al., 2015; Louie et al., 2016; Goswami et al., 2017). To understand how NMs behave in ecosystems and to determine their toxicity and fate, we must first understand the life cycle and mechanism of NMs transformation processes upon their release into environmental compartments and their interaction with the surrounding environmental components (Figure 1).

### 2.1 Chemical transformations

Dissolution is a key chemical transformation process driven by the release of water-soluble molecules or ions from NMs. The equilibrium solubility (amount of dissolved matter) and the kinetics of particle dissolution (rate of solubility) of NMs will affect their toxicity, behaviour and environmental fate. In general, NMs that readily dissolve were more toxic than poorly soluble NMs, as once bioaccumulated in organisms they undergo rapid dissolution, leading to oxidative stress from the release of reactive oxygen species (ROS) via a so-called trojan horse mechanism. Sulfidation is a major transformation process for many metal NMs, particularly when enhanced concentrations of sulfide are present, such as the ones found in sub-oxic or anoxic sediments or in some parts of wastewater treatment plants (Lead et al., 2018). Sulfidation can reduce solubility, and change particle size and surface charge of NMs and in most studies, the sulfidation of NMs reduces their toxicity (Devi et al., 2015; Starnes et al., 2015). Photochemically induced reactions are another main driver of NM transformation, including photolysis, photo-oxidation and photo-catalysis, with sunlight playing an important role in the dissolution of NMs (Goswami et al., 2017; Kansara et al., 2022; Xu et al., 2023).

### 2.2 Physical transformations

The physical transformation of NMs leads to alterations in their stability when interacting with environmental components, due to changes in the local ionic strength and pH or due to interactions with sediments and NOM, and are affected by other physical parameters such as sunlight exposure and temperature (Kansara et al., 2022). Agglomeration and sedimentation/deposition are the main physical transformation processes that NMs undergo once released into the environment (Abbas et al., 2020). Agglomerates are particle clusters held together by electrostatic reactions or chemical bonds, they cluster together due to the attractive forces between particles, and this can occur during use, production, storage or upon release of NMs into the environment (Hartmann et al., 2014). The surface area to volume ratio, and thus NMs reactivity, is reduced by the agglomeration process, which will affect their toxicity, transport in porous media, reactivity, sedimentation and uptake by organisms (Lowry et al., 2012). Lead et al. (2018) demonstrated in their review that although many studies showed that agglomeration usually reduces the bioavailability of NMs, however, in some cases it can enhance bioaccumulation by increasing the ingestion rate or by making the particles size more accessible (Martinez et al., 2022). Abbas et al. (2020) also highlighted that at realistic environmental concentrations, homoagglomeration (single NMs only) was proven to be quantitatively unimportant, suggesting that heteroagglomeration (mixture of NMs/particle types) could be more important due to the higher environmental concentrations of natural colloids such as clay. Agglomeration leads to a reduction in NMs number concentration in water or soil suspensions favouring the deposition of agglomerated large particles (Abbas et al., 2020). Larger, denser, particles tend to settle faster than smaller particles and therefore gravitational settling will reduce NM migration (Hartmann et al., 2014; Yin et al., 2015; Giorgi et al., 2021).

### 2.3 Biological and macromolecular NM transformations

The major biologically mediated transformation processes that NMs undergo upon their release into natural environments are eco/bio-corona interactions and biodegradation (Abbas et al., 2020). Biomolecules are readily adsorbed onto NM surfaces in ecosystems, and NMs that are taken up by biological organisms can be transformed upon their interaction with biomolecules (Lowry et al., 2012; Xu et al., 2023). Additionally, metal ions can be transformed into NMs due to the presence of functional groups and reductive enzymes, for example, metal ions can be transformed into their corresponding NMs by a reducing agent, such as ascorbic acid (C_6_H_8_O_6_), which occurs naturally (Abbas et al., 2020).

The process of biodegradation is driven by the ability of microorganisms to decompose an organic substance. The interaction of NMs with microbes, extracellular polymeric substances (EPS) and extracellular enzymes determines their relative significance and rate of the biodegradation processes. Biodegradation of NMs core components and surface coatings is a possible transformation pathway of NMs in surrounding environments, particularly for carbon-based NMs including fullerene and carbon nanotubes (CNTs) (Abbas et al., 2020). The biodegradation of organic surface coatings is relevant to all types of manufactured NMs and to MPs, and the gut microbiota of aquatic organisms are likely to play a key role (Nasser et al., 2020). Biomodification is an additional process that can affect the fate and toxicity of NMs, which includes processes that are indirectly mediated by organisms after NMs are taken up by the organisms (Kelpsiene et al., 2022).

In natural environments organisms such as *Daphnia* produce and secrete a variety of tissue extracts that contain biomolecules (e.g., proteins and polysaccharides). These secreted biomolecules can form coronas on NMs and MPs as the biomolecules adsorb onto the surface of the particles. These coronas are dynamic in nature, and result from the adsorption of different types of available proteins, metabolites and polysaccharides, which also exchange between bound and free forms (Westmeier et al., 2016; Grintzalis et al., 2019; Kelpsiene et al., 2022). Environmental and biological constitutes that have a molecular weights spanning from 10 to 2,000,000 Da adsorb onto NMs surfaces forming the eco-corona, and affecting the stability, identity, uptake and toxicity of NMs towards *Daphnia* (Nasser et al., 2020). The adsorbed proteins can also facilitate NMs entry into cells through the receptor-mediated endocytosis process (Lowry et al., 2012). Furthermore, identifying proteins present on the surface of NMs can provide important insights into their biological interactions, including uptake and which mechanistic pathways are induced by NM exposures as part of an ecotoxicity assessment (Ellis and Lynch, 2020; Wheeler et al., 2021). Naturally occurring biomolecules (e.g., natural organic matter (NOM), humic substances) also play a major and similar role when interacting with NMs. Therefore, the fate and behaviour of NMs is highly dependent on understanding the characteristics of these macromolecular processes. Interactions with NOM and macromolecules can increase NMs persistence in aquatic systems (Wang et al., 2016).

### 2.4 Environmental testing conditions—bridging the gap between field and laboratory

Current guidelines for NMs ecotoxicology tests do not prioritise the use environmentally transformed NMs in environmentally representative waters, for example, natural waters that are complex matrices containing natural organic matter and other biopolymers, the concentrations and compositions of which go through various environmental changes which can impact the physiochemical properties and toxicity of the NMs they interact with. Estimates for NM toxicity based on simplified salt only media thus under or over-estimate the impacts of NMs by not testing the appropriate NMs forms (Selck et al., 2016; Ouyang et al., 2022). To allow for comparisons between toxicity studies, the Organisation for Cooperation and Development (OECD) promotes the use of a fully defined testing medium for exposures in ecotoxicity testing. As with many biological elements, there is a narrow concentration range between deficiency and toxicity of key elements which needs to be carefully considered, as the testing medium needs to be suitable for the test species (i.e., both algae and *Daphnia* during chronic testing). Medium composition can also impact the toxicant in question, for example, when testing metal toxicity, it is important to ensure there are no chelating agents, such as EDTA, present that would react with the metals and therefore change the metal bioavailability during the exposure. Media such as the OECD ‘M4’ and ‘M7’ can be modified by removing the EDTA, or an alternative medium can be used that contains no chelating agents (OECD, 2004).

The OECD 202 and 211 tests (along with most current test guidelines) were designed for testing of chemical toxicants, and as such, modification of medium for emerging pollutants such as NMs and MPs, with their large reactive surfaces, and more environmentally realistic testing scenarios is needed (Giusti et al., 2019; Ellis et al., 2020a; Nasser et al., 2020). This includes the potential addition of NOM, which is the decaying plant and animal matter present in natural waters and soils, and has been described as containing varying fractions of humic acids, fulvic acids, polymeric substances and a hydrophilic fraction, and which has been widely reported to have strong absorption to colloidal materials (Afshinnia et al., 2018; Tayyebi Sabet Khomami et al., 2020), or the use of medium conditioned by pre-filtration through *Daphnia* guts (or other relevant organisms used for toxicity testing, including oysters, worms, etc.) which is often termed “conditioned medium,” for example, (Nasser and Lynch, 2016). Although the addition of NOM is not recommended by the OECD due to its heterogenous nature, NOM can act as a stabiliser for NMs within the testing medium, preventing agglomeration of the NMs and therefore maintaining the bioavailable fraction. On the other hand, NOM can sorb chemicals in test solutions which can ultimately affect the fate and bioavailability of the toxicants within the test solutions by decreasing the concentration in the dissolved phase and changing the exposure pathway. The addition or exclusion of NOM is therefore test dependent and should be considered during the toxicity test design stage.

Given the role of standardised tests in ranking the toxicity of chemicals, including NMs and MPs, as well as the use of data from *Daphnia* toxicity assays for environmental modelling and establishment of threshold levels for pollutants, a deeper understanding of the inherent variability in the test systems is needed. Similarly, the standardised test media has been developed to optimise the test population health, which does not take into consideration any deficiencies in species health or fitness that occur due to natural environmental variation and adaption to the environment. Utilisation of laboratory cultured daphnids, whose conditions are optimised for health and fitness and where there is no competition for food and no predation, could mean that we underestimate the potential toxicity of chemicals to real environmental populations, especially when looking at sublethal toxicity markers such as growth and reproduction due to the lack of variability in other parameters such as temperature and food availability. However, examples emerging in the literature are showing that the “as engineered pristine” NM have fewer toxic consequences in environmentally realistic medium compared to the standard culture media used in standard toxicity testing (Schultz et al., 2018; Ellis and Lynch, 2020; Schultz et al., 2020). Thus, standardised *Daphnia* tests following the OECD protocols overestimate NMs toxicity, which can be resolved through using environmentally transformed NMs in representative natural water compositions (Nasser and Lynch, 2019; Ellis and Lynch, 2020; Nasser et al., 2020). Conversely, wild daphnids from the field are believed by aquatic toxicologists to have a higher resistance towards pollution compared to *Daphnia* that have been cultured in the laboratory over long periods (Abdullahi et al., 2022; Eastwood et al., 2022). This is due to the exposure of wild *Daphnia* to a much wider range of natural stressors than their lab-cultured equivalents where conditions are closely controlled, leading the wild daphnids to have resistance towards pollutants, decrease of water quality and competition for a limited of food supply, resulting in a greater overall “fitness” and ability to survive in changing environments (Barata et al., 2000). Taking this into consideration, a precautionary approach should be applied when applying lab observations to field studies and *vice versa*.

According to Brans et al. (2018), human-induced and natural stressors induce changes in energy metabolism and stress physiology in populations of a wide array of species. Urbanization is a pervasive process with 476,000 ha of arable land are lost annually by the expansion of urban areas (World Resources Institute, 1996; Grimm et al., 2008). Urbanization alters both biotic and abiotic ecosystem properties within and surrounding the urban centre (Miles et al., 2019; Ruas et al., 2022). Differential selection of stress-coping mechanisms results from stressful environments like those found in cities. For instance, city ponds are exposed to the urban heat island effect and receive polluted run-off, with the result that several stressors may act together and affect the life traits of organisms inhabiting these ecosystems, which might acquire genetic differentiation for physiological traits enabling them to cope better with higher overall stress levels (Pavlaki et al., 2014). Evidence from 62 *Daphnia* genotypes from replicated urban and rural populations in garden ponds revealed that urban *Daphnia* have significantly higher concentrations of total body fat, proteins and sugars than their rural counterparts (Brans et al., 2018) highlighting that environmental conditions contribute to *Daphnia* fitness. This can be further explored through study of acclimation, also called adaptation, resistance, or tolerance, which has been defined as the ability of organisms to cope with stress, either natural such as temperature changes, salinity variation, oxygen level fluctuations, and plant toxins or chemicals arising from anthropogenic inputs of many different classes of contaminants into the environment (Biagianti-Risbourg et al., 2013; Akbar et al., 2022). The capacity of physiological adaptation or acclimation toward a stressor is related to the stress syndrome. Physiological acclimation to toxicant conditions also depletes energy reserve levels (Biagianti-Risbourg et al., 2013). For example, in *Daphnia* organisms pre-exposed to zinc (and having acquired a tolerance toward this metal) did not mobilize their energy reserves further following a laboratory exposure to zinc (0.1 and 1.0 μM) compared with non-exposed animals (Canli, 2005).

Therefore, the culturing history of the *Daphnia*, or other test species, in addition to the environmental conditioning of the NMs or MPs in the exposure medium can have a significant impact on the toxicity response and the overall impact on the ecosystem, and this should be considered at the test design stage to determine the adequate levels of comparability and environmental relevance of the exposure.

## 3 The development of *Daphnia* as a model organism: lifecycle, reproduction and multigenerational approaches

One of the most important issues to address in toxicological testing is how exposure, whether it be acute or chronic, impacts the organism and the subsequent effects to their offspring. When environmental conditions deteriorate, for example, due to an influx of environmental pollution or predator stress, daphnids can develop different phenotypes and can switch from clonal to sexual reproduction (Colbourne et al., 2011; Eastwood et al., 2022).

Under favourable environmental conditions (e.g., within the optimal range shown in Table 1), *Daphnia* reproduce parthenogenetically (clonally). Parthenogenesis is a type of asexual reproduction in which the offspring develops from unfertilized eggs. Female *Daphnia* produce genetically identical daughter clones, which are released from the brood pouch as neonates (Ebert, 2005). The reproduction process continues while the environmental conditions continue to support their growth. *Daphnia* can change to sexual reproduction under stressful conditions, such as overcrowding, low food, toxicant exposures or variations in abiotic factors such as temperature and pH (Alekseev and Lampert, 2001; Abdullahi et al., 2022). This results in the development of ephippia, or resting eggs, that can remain dormant in the sediment for long periods of time (years) and may hatch when conditions improve (Eastwood et al., 2022).

**TABLE 1:** Recommended conditions for optimal culture growth of *Daphnia* as outlined in the OECD test guideline for acute toxicity (OECD 202).

Factor	Optimal range
pH	6–9
Temperature	18–22°C
Dissolved oxygen	>6 mg/L, ideally at saturation
Water Hardness	140–250 mg CaCO_3_/L
Light/dark cycle	16 light/8 dark

As a consequence of NMs induced stress, the genetic processes are altered, which can be easily monitored by identification of epigenetic (heritable from one generation to the next) changes in subsequent generations. These changes are due to modifications of the histone proteins of chromatin and DNA methylation, which results in altered gene expression (Feil and Fraga, 2012). This makes *Daphnia* an ideal model organism for studying the effects of NM toxicity, as epigenetic developmental programs can be used to monitor the effects in the offspring as hereditary traits (Asselman et al., 2017). Having the ability to monitor the offspring after NM parental exposure provides invaluable information regarding the molecular events that occur for survival, growth, reproduction, and adaptation to change (Abdullahi et al., 2022).

An advantage of using multiple generations that follow a germline after parental exposure, is that the genes expressed at an early stage of exposure might not be the same genes as those directly associated with phenotypic effects over a chronic exposure time scale. Therefore, capturing the phenotypical events in the offspring will also identify the longer-term causable effects (van Straalen and Feder, 2012). Multigenerational studies also help to demonstrate how maternal effects of exposure to what is considered a sub lethal concentration, results in a trade-off between growth, reproduction, and survivorship over all generations, which ultimately defines the natural selection of the strongest surviving daphnids.

Several multigenerational studies using *Daphnia*, in the presence of either chemical or NM exposure have each reported an increased toxic effect in the immediate post-parental exposure generations compared to the exposed parental generation (Arndt et al., 2014; Jeong et al., 2016; Kim H. Y. et al., 2017; Ellis et al., 2020b). A study investigating the species difference between *D. magna*, *pulex* and *galeata* over five generations exposed to silver nanoparticles demonstrated that NM exposure had the most negative effects on the first generations, with notable changes between increased toxicity and tolerance in the subsequent generations (Völker et al., 2013). The altered toxicity in the latter generations provides evidence that the ecological risk and safety assessments underestimate NM toxicity using only single generation acute and chronic tests. Ellis et al. (2020b) also identified that the transgenerational responses of multiple germlines showed a direct link with maternal exposure time to ‘sub-lethal’ effect concentrations of NMs (identified from standard OECD acute toxicity tests which chronically presented as lethal) including increased survival and production of males for sexual reproduction in the subsequent germlines (Ellis et al., 2020b). Multigenerational studies using only pristine engineered NMs have manifested adverse toxicological outcomes in multiple generations post maternal exposure to *Daphnia* that could not have been predicted from the single standard 1-generation reproductive studies (Ellis et al., 2020b; Ellis and Lynch, 2020; Karatzas et al., 2020). Collectively, the research demonstrates the importance of updating standard toxicity testing to reflect scientific advances and increase trust in regulation by monitoring the effects in the transgenerational germlines (Jeong et al., 2016; Ellis et al., 2020b; Nederstigt et al., 2022).

## 4 Feeding behaviour of daphnids and bioaccumulation potential of NM

*Daphnia* are filter-feeders that have the ability to ingest particulates of up to 50 μm in size through mechanical sieving mechanisms (Geller and Müller, 1981). *Daphnia* mainly feed on phytoplankton, such as green algae (considered as a high-quality food, such as *Scenedesmus* sp.), bacteria and organic detritus (considered as a low-quality food). However, their non-selectivity in the uptake process increases the bioaccumulation of environmentally unfriendly materials along the higher trophic levels (Schwarzenberger and Fink, 2018). Therefore, *Daphnia* are likely recipients of contaminants, including NMs and MPs and, as primary consumers, they are vital for energy transfer in the food chain (Martinez et al., 2022; Yin et al., 2023).

### 4.1 Nano-intestinal interactions and gut chemistry

The gut luminal chemistry is of particular interest to comprehend the fate and adverse effects of NMs on the organism after NM ingestion. The pH (6.8–7.2), ionic strength (e.g., Na^+^, Ca^2+^, and Mg^2+^), the presence of NOM, the cuticle chemistry, and redox chemistry are factors that interfere in the absorption of particulates in the gut, and they act as barriers in NMs exposure during uptake from the gut into the tissue (Van Der Zande et al., 2020). From the external environment to the gut, NMs are likely to acquire an eco-corona which is generally expected to reduce their toxicity (Ekvall et al., 2021), although if the acquired corona results in some particle agglomeration the particles may become a more attractive food source and thus be taken up to a greater extent resulting in increased toxicity (Nasser and Lynch, 2016). Depending on their location in the intestine, NMs can acquire an unique eco-corona profile (Chetwynd et al., 2020; Cui et al., 2020), and undergo different transformations, for example, a pH-dependent dissolution (Cao et al., 2022). Due to the particularities of NMs, they are normally dispersed in the luminal liquid, rather than dissolved, and a wide diversity of macromolecules (solid-phase food, exudates, digestive enzymes, and proteins) present in or from the external environment can also be considered as additional colloidal components that can contribute to its dispersibility or not (Nasser et al., 2020; Van Der Zande et al., 2020). The composition of the digestive tract and the interaction forces between NMs and gut lumen matrix determine NMs bioavailability, potentiating or mitigating NM toxicity to daphnids (Cui et al., 2020). Consequently, the physicochemical characteristics of NMs and natural biological constituents must be studied in terms of their colloidal chemistry to determine their colloidal behaviour and impacts on daphnids’ physiology (Christenson, 1984).

### 4.2 Enzymes as biochemical markers of nanotoxicity

The median effective concentration leading to immobility (EC_50_) or lethality (LC_50_) are the OECD standardised endpoints considered in acute toxicity assessment (OECD, 2004), while body burden, reproductive, and growth rate are the end points for the chronic assessment (OECD, 2012). However, these apical endpoints are less sensitive than the biochemical ones (De Coen and Janssen, 1997). To enable a better understanding of the adverse outcomes on food metabolism and stress response, we need to identify suitable biomarkers for ecotoxicity (Schwarzenberger and Fink, 2018). Biochemical markers can work as early indicators (sub-lethal toxicological effects) of perturbance on organisms metabolism, resulting in alterations in enzyme activity or expression, and can be identified using enzyme assays (Galhano et al., 2020; Galhano et al., 2022), or a range of omics techniques (Taylor et al., 2018; Zhang et al., 2018; Bhagat et al., 2022), respectively. Here, we summarise the main findings reported for two major enzymes classes (digestive and antioxidant) from *Daphnia* nanoecotoxicology studies (Table 2).

**TABLE 2:** Effects of exposure of *D. magna* to NMs on the activity and/or expression of antioxidant and digestive enzymes.

NMs	Dose (mg L^−1^)	Co-exposure with	Exposure time (days)	Antioxidant enzymes		Digestive enzymes		EC_50_/LC_50_ (mg L^−1^)	References
				With effect	No effect	With effect	No effect		
TiO_2_	0, 1, 5, 10	-	2	↑CAT, GST, GPX	SOD	-	-	Non-toxic	Kim et al. (2010)
TiO_2_ ^b^	1	-	2	↑CAT	SOD	↓esterase	cellulase, trypsin, amylase	-	Fouqueray et al. (2012)
			21	-	CAT, SOD	↓trypsin, amylase, esterase	cellulase	-	
	1.10^–2^	-	2	-	CAT, SOD	↓amylase	cellulase, trypsin, esterase	-	
			21	-	SOD, CAT	↓amylase, esterase	cellulase, trypsin	-	
C_60_	0, 5, 20	-	1	↑SOD	-	↓trypsin, amylase, cellulase, b-galactosidase	-	-	Lv et al. (2017)
			2	↑SOD	-	-	-	-	
			3	↓SOD	-	-	-	14.9 ± 1.2/16.3 ± 0.8	
MPA -Au	1	-	1	↑GST	CAT	-	-	Non-toxic^c^	Qiu et al. (2015)
			21	↓CAT	GST			Non-toxic	
PAH -Au	5.10^–3^	-	1	-	GST, CAT	-	-	-	
			21	-	GST, CAT			Toxic	
MPA -Au	5.10^–2^	-	1	-	GST, CAT	-	-	Non-toxic^c^	Dominguez et al. (2015)
	1.10^–2^			↑CAT	GST				
PAH -Au	5.10^–2^	-	1	↑GST	CAT	-	-	Toxic^c^	
	1.10^–2^			↑CAT	GST				
TiO_2_	2	Cu^2+^	3	↑↓CAT, SOD, ↓Na^+^/K^+^ ATPase	-	-	-	-	Fan et al. (2012)
QDs-indolicidin	1.5^a^	-	3, 9	↓SOD, CAT, ↑GST	-	-	-	Non-toxic	Falanga et al. (2018)
			15, 24	↑GST, CAT	SOD				
ZnO	1.10^–1^	-	1, 3, 7, 14	↓SOD, GST, CAT	-	-	-	1.04^c^/-	Chen et al. (2017)
C_60_ ^b^	0.5–2	-	21	-	-	↓amylase, trypsin, lipase	-	-	Tao et al. (2016)
TiO_2_	1	Cu^2+^	2	↓SOD	Na^+^/K^+^ ATPase	-	-	-	Liu et al. (2015)
ZnO	0.8, 1.1	-	3	↓ GST	-	-	-	-	Mwaanga et al. (2014)
CuO	0.8, 1.1	-	3	↓ GST	-	--	-	-	

^a^ Concentration unit: nM.

^b^ NMs, ingested from dietary route.

^c^ 48 h of exposure.

↑ increase of enzyme activity/gene expression related, while ↓ implies decrease.

#### 4.2.1 Digestive enzymes (food metabolism)

In addition to the mechanisms involved in the digestive process mentioned earlier, digestive enzymes play an important role in the metabolism of ingested food, breaking down food particles and increasing the efficiency of digestion. Overall, several enzymes are secreted to metabolize proteins such as trypsin and chymotrypsin, sugars such as cellulase, α-amylase and β-galactosidase; and lipids such as esterase (Lv et al., 2017). When exposed to NMs and/or pollutants, their activity is changed to maintain the homeostasis of organism’s metabolism. However, depending on the dosage level which they are exposed, these compounds can affect digestive physiology and food metabolism in *Daphnia* (Qi et al., 2022). NMs were shown to target mainly intestine epithelium and peritrophic membrane (Mattsson et al., 2016). Recent evidence depicted that NMs can penetrate cell membrane without disrupting it, and be endocytosed (Santo et al., 2014). NMs physicochemical characteristics (e.g., shape, size, and surface chemistry) are determinant to the chemical transformations they undergo in the digestive tract of daphnids and later elicited biological responses (Liu et al., 2019).

An inhibition on the activity of amylase and esterase in the treatment with a low and higher concentration of TiO_2_ NMs in acute toxicity assessment was observed, and this effect was even more evident during a chronic assay. Exposure to the NMs has shown to affect the nutrition, growth, and reproductive processes in daphnids (Fouqueray et al., 2012) and this was demonstrated by a dose-dependent decrease in the enzymes’ activity after exposure to fullerene (C_60_) (Lv et al., 2017). In another work, the activity of enzymes was monitored over days, which confirmed a reduction of their activity in *D. magna* (Tao et al., 2016). In *D. pulex*, zinc oxide NMs (ZnO NMs), bulk (ZnO), and ionic species (Zn^2+^) dysregulated the expression of genes related to chymotrypsin, carboxypeptidases, and serine protease enzymes (Lin et al., 2019). Disruption of intestinal structure of daphnids after interaction with stressors commonly impacts on energy acquisition and causes high metabolic costs, via reduction of the available energy reserves (carbohydrates, lipids, and proteins), to keep the basal metabolism. The adsorption of NMs to active sites or surface of trypsin enzyme was suggested as a possible mechanism to inactivate digestive macromolecules by a comparable experimental and theoretical study (Zhang et al., 2014).

#### 4.2.2 Antioxidant enzymes (oxidative stress)

Daphnids initially respond to the intake of foreign materials (NMs and contaminants) by producing ROS, which results in oxidative stress. Prolonged exposure to these stressors can lead to lipid peroxidation, protein inactivation, and DNA damage. Antioxidant enzymes modulate their activity to reduce the damage caused. Superoxide dismutase (SOD) is the first defence line in detoxification that produces the substrate for catalase (CAT) to metabolize. Then, CAT, glutathione-s-transferase (GST), and glutathione peroxidase (GPx) remove harmful metabolites generated from this process transforming them into less toxic compounds, such as water and oxygen (Galhano et al., 2022). Proteins associated with oxidative stress response can be found in the NM eco-corona, giving insights into the mechanistic pathways associated with NM toxicity (Nasser and Lynch, 2016; Fadare et al., 2019; Ellis and Lynch, 2020).

Similarly, *Daphnia* exposed to C_60_ (fullerenes) increased SOD activity after 48 h, and decreased after 72 h, indicating oxidative stress damage and possibly the beginning of lipid peroxidation since simultaneously a dramatic increase of malondialdehyde (MDA) was obtained (Lv et al., 2017). Increasing dose of TiO_2_ NMs exposed to daphnids augmented the activity of CAT, GST, and GPx but no effect was observed for SOD (Kim et al., 2010). CAT response was similar to those observed for GST and GPx, but in long-term exposure to the NMs, their activity recovered due to acclimation (Fouqueray et al., 2012). In contrast, ZnO NMs increasingly inhibited the activity of enzymes as exposure duration increased. Similarly, the genes corresponding to SOD and GST were upregulated initially and later downregulated, while MDA content increased over time, indicating that the detoxification was overwhelmed and possibly led to GST inactivation (Mwaanga et al., 2014; Chen et al., 2017). Exposure to quantum dots (QDs) functionalized with indolicidin (an antimicrobial peptide) also affected enzyme efficiency, but SOD adapted to the stress condition, while CAT was greatly induced after 15 days and GST activity slightly increased during period of exposure (Falanga et al., 2018).

The role of surface chemistry on enzyme activity was investigated using gold NMs (AuNMs). Negatively charged AuNMs (MPA-AuNMs) caused a significant increase in *gst* expression, related to GST, while no effect was noted from positively charged (PAH-AuNMs) after 24 h (Qiu et al., 2015). However, in another work, exposure to PAH-AuNMs, induced *gst* expression compared to MPA-AuNMs (Dominguez et al., 2015). Both studies considered that the toxicity may be associated with the AuNMs colloidal behaviour, because PAH-AuNMs were more stably dispersed in the test medium than the MPA-AuNMs, they were more bioavailable to be absorbed and cause damage to the daphnid gut. PAH-AuNMs was slightly toxic even at a low exposure dose (5 μg L^−1^) and increased significantly at 10 and 50 μg L^−1^ (Bozich et al., 2014). The contrasting responses in these studies may have resulted from the distinct exposure conditions (i.e., the concentration used).

Combining NMs with inorganic or organic contaminants is an attractive approach to investigate the oxidative damage triggered in a real-world-like scenario (Galhano et al., 2022). For example, exposure to TiO_2_ NMs in a range of concentration (from 10 to 100 μg L^−1^) of copper (Cu^2+^) resulted in induction of CAT, reaching a maximum of 10 and 20 μg L^−1^ in the presence and absence of the NMs respectively. Inhibition of CAT occurred as the dose of Cu^2+^ increased (Fan et al., 2012). Similar behaviour was described for SOD, but no statistical difference was found between the exposure in the presence and absence of TiO_2_ NMs, meaning that the presence of NMs had no additional effect on the activity of this enzyme. However, an inhibitory effect was observed in Na+/K+ ATPase transporter in mixture conditions compared to the condition without TiO_2_ NMs. With respect to the mortality rate, the co-exposure of TiO_2_ NMs with Cu^2+^ increased the toxicity compared to the treatment which organisms were exposed to Cu^2+^ only (Fan et al., 2012). In another study, TiO_2_ NMs with varied percentage of free {001}facets combined with Cu^2+^ reduced SOD activity, but just a slight decrease was observed in Na+/K+ ATPase activity and no change in this transporter activity in the single exposure condition to the NM (Liu et al., 2015). In Mwaanga et al. (2014) work, NOM was added to the test medium and reduced the inhibitory effect of ZnO and CuO NMs on GST activity, possibly, by diminishing the accessibility of NMs to adsorb GST.

Galhano et al. (2020) integrated an individual and subcellular level approach to effectively assess the toxicity of TiO_2_ NMs (12.5–100 μg L^−1^ Ti) or AgNPs (25–125 μg L^−1^ Ag), under environmentally relevant conditions, i.e., transformed NMs. The authors observed significant alterations in the activity of GST and CAT mainly after exposure to AgNPs dispersed in wastewater compared to test water. Later, Galhano et al. (2022) evidenced that only in the case of AgNPs dispersed in the wastewate and effluent was SOD activity decreased. The differences in the enzymatic activity of antioxidant enzymes under the conditions of exposure were indicated as resulting from the difference in the physicochemical characteristics of the TiO_2_ NMs under the different exposure conditions (7.8 and 4,761.4 μg L^−1^ Ti in water, 6.3 μg L^−1^ Ti in wastewater-borne, 17.3 and 5,467.5 μg L^−1^ Ti in effluent) and AgNPs (81.7 and 105.4 μg L^−1^ Ag in water, 30.3 μg L^−1^ Ag in wastewater-borne, 56.4 and 80.5 μg L^−1^ Ag in effluent), the complexity of the matrices and the aging of the effluents used (Galhano et al., 2022). Stable agglomeration of TiO_2_ NMs may have reduced their bioavailability. Interestingly, the author also observed a toxicity coming from the background effluent that proved to be relevant and should be considered in further studies (Galhano et al., 2022).

### 4.3 Challenges and perspectives for enzyme activity studies in *Daphnia*

Most studies on nanobio-interfacial interactions in the gut have been carried out on animal models, and cultures of intestinal cells from invertebrates are not yet available, representing a great challenge for the advancement of research on the underlying mechanisms of absorption and bioavailability of NMs in invertebrates. The small size of daphnids and sample contamination (e.g., mucus and carapace) are the main limiting factors that influence data collection (Mattsson et al., 2016; Van Der Zande et al., 2020). Besides investigating nano-intestinal epithelial cells interaction, understanding NMs interactions with *Daphnia* gut microbiota are necessary, since ingested NMs and other stressors change the composition and functioning of microorganisms that inhabit the gut (Akbar et al., 2020; Cui et al., 2020). The alteration of life history traits have also been shown to mediate the toxicity response (Li et al., 2019), however, the understanding of life history and gut microbiome influence NM and MP toxicity are still in their infancy (Li et al., 2019; Akbar et al., 2020; Varg et al., 2022).

Despite biochemical markers being promising tools for early aquatic toxicity assessment (Michalaki et al., 2022), the biochemical and physiological responses generally do not correlate and sometimes there is no consistency in the results. Therefore, it is essential to push the scientific community to harmonise experimental procedures to produce reliable data, following the Findable, Accessible, Interoperable, and Reusable (FAIR) principles (Wilkinson et al., 2016), which will later allow linking physiological responses with molecular patterns and (sub) organismal responses and facilitate computational modelling and predictive (nano) toxicology. This will allow computational modelling of current data, identification of biomarkers that predict adverse outcomes and decision making for regulatory purposes (Taylor et al., 2018).

### 4.4 Bioaccumulation of NMs in aquatic food webs and quantification of particle uptake

**FIGURE 2:**
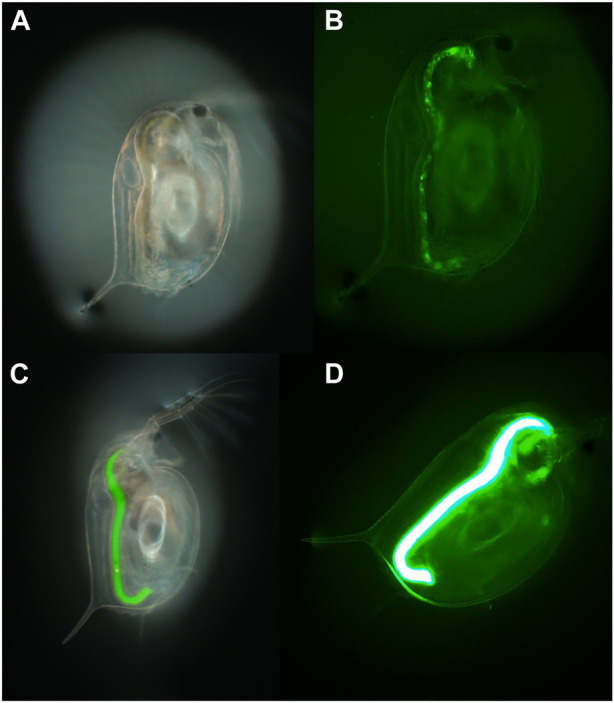
An example of the use of fluorescence to determine uptake of 1–5 µm polyethylene MP particles by *D. magna* visualised in two daphnids exposed to different concentrations of the MPs (50 and 500 mg/L, respectively) for 24 h. **(A,C)** brightfield, and **(B,D)** fluorescence imaging of the first and second daphnids, respectively. Taken with an Olympus optical microscope with a Green Fluorescent Protein (GFP) filter cube and dichroic mirror and a DP74 colour camera and viewed using CellSens software (×70 magnification).

Uptake and bioavailability studies are significant for studying the behaviour of NMs in natural environments and for linking the biological effects to the environmental chemistry of NMs (Lead et al., 2018). In addition to microorganisms, crustaceans such as *Daphnia* are involved in the degradation of organic matter and nutrient recycling, working as shredders in natural ecosystems and acting as pivotal components of the food web (Ebert, 2005). Further to already being an established model species for regulatory testing, *Daphnia* are an advantageous model for NM and MP toxicity testing due to their clear body which allows visualisation of the uptake and potential retention of NMs and MP within the *Daphnia* gut. Fluorescently labelled industrial beads have been widely used to undertake initial toxicity assessments of MPs, which uses the fluorescence as a proxy for the MPs to theoretically determine uptake, translocation, and potential storage in the organism’s tissue (Figure 2) (Rosenkranz et al., 2009). However, the potential for dye to leach from the beads can confound the results, as dyes can be retained in the lipid deposits and other tissues within the *Daphnia* leading to incorrect tracing of the MPs and to misreporting of translocation of MPs in cases where this has not occurred (Schür et al., 2019). For example, Nile red is often used as a stain to identify MP in environmental samples, but this dye is also widely used for lipid staining within *Daphnia* which can lead to overestimation of internalised MPs concentrations and retention. Furthermore, the change in internal biological conditions (such as pH) can affect the fluorescent signalling from the dye which can significantly impact the results or could further impact the dye leaching from the particles (Triebskorn et al., 2019; Davis et al., 2020).

**FIGURE 3:**
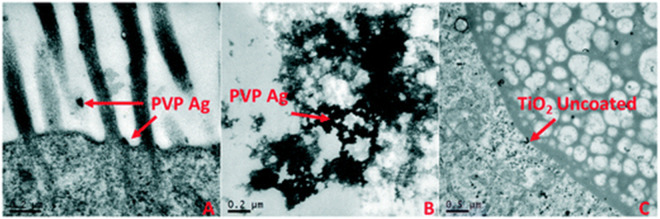
An example of the use of TEM images to determine the accumulation of NM in *Daphnia* guts and potential translocation. Uptake and localisation of **(A)** freshly dispersed PVP-coated Ag NMs in HH combo medium showing microvilli interactions, **(B)** freshly dispersed PVP-coated Ag NMs in HH combo found in the gut lumen space; and **(C)** freshly dispersed uncoated TiO_2_ NMs in HH combo, showing evidence of NM translocation in the bush border. Reproduced from (Ellis and Lynch, 2020) with permission from the Royal Society of Chemistry.

Quantifying the internalised concentration of particles during exposures can vary depending on the material type but is very useful data to collect as the internalised concentration can be used to more accurately determine a dose response relationship. Internalised concentration, or body load, is needed for Toxicokinetic-Toxicodynamic (TK-TD) modelling such as Dynamic Energy Budgets (DEB) which can link several aspects of life history trait observations to potential changes in the population distribution. Some of the more widely used methods for visualisation of particle accumulation and damage are transmission electron microscopy (TEM) to determine the uptake, potential translocation and retention of particles within organisms tissues as per the example shown in Figure 3 (Ellis and Lynch, 2020) and ICP-MS analysis for quantification of metal, or metal doped, particles in tissue (Schmiedgruber et al., 2019; Ellis and Lynch, 2020).

## 5 *Daphnia* genome and key NMs toxicity pathways

Direct investigations of how NMs are associated with adverse outcomes and disease in humans are very limited (Lima et al., 2022), mainly due to the issues around how chemicals including NMs are regulated under existing chemical frameworks and the ethical limitations of testing directly on humans (Hansen, 2017; Oksel Karakus et al., 2021). Due to the shared mammalian biology, information safeguarding human health relies on the toxicological information from invertebrate (mice and rat models) *in vivo* studies to protect human health (Festing and Wilkinson, 2007; Franco, 2013), as well as fish and algae, which have been traditionally used to set regulatory limits to safeguard environmental health (Brunner et al., 2009; Kaltenhäuser et al., 2017).

The principles of the 3Rs (Replacement, Reduction and Refinement) were developed over 50 years ago providing a framework for performing more humane animal research (https://nc3rs.org.uk) (Burden et al., 2017; Sneddon et al., 2017). Advancement of animal genome research over the last 20 years has led to significant understanding of the relationship between simple organisms and their changing environment (van Straalen and Feder, 2012; Stracke and Hejnol, 2023). Moreover, the advancement in evolutionary developmental biology and ecological functional genomics, has identified a possibility to reduce the use of animal experimentation using simple model organisms including invertebrates, due to the large amount of the genome that is conserved across species. Genomics is used to identify genetic variation under natural selection (Mitchell-Olds and Feder, 2003; Leinonen et al., 2013; Grummer et al., 2019) in model test organisms, which are accessible to both laboratory and field studies along defined environmental gradients (Spanier et al., 2017). Furthermore, comparative studies into the evolution and conservation of genes and genomes, provides significant information on genetic diversity and similarities among major groups of organisms from simple organisms to larger invertebrates (Ros-Rocher et al., 2021).

### 5.1 *Daphnia* genome

Characterisation of *Daphnia* genomes enables the progress of molecular ecotoxicology for evaluating pollutants and NMs impacts, by analysing molecular pathways related to their defence mechanism response (Lee et al., 2019). The complete genome sequence of *D. pulex* (Colbourne et al., 2011) and *D. magna* have been elucidated but the search for key gene families related to stressors exposure is ongoing through screening of likely molecular biomarkers (Orsini et al., 2016). Identification of genomic expression profiles enables understanding of how genes are regulated under different ecological conditions and how these expressions are linked to phenotypic change (Rozenberg et al., 2015; Hales et al., 2017; Ros-Rocher et al., 2021). Phenotypical variation is understood to be largely due to gene and environment interactions that were shaped by evolution, or by environmental stress that predictably disrupt the normal functioning of genes (Hodgins-Davis and Townsend, 2009; Moyerbrailean et al., 2016; Alexander-Dann et al., 2018).

The expression profile (when compared to a non-treated control organism) of all genes that are expressed in response to a particular initiating event, is called a “molecular phenotype.” The transcription of the “molecular phenotype” is based on the evolutionary history of populations (Ravindran et al., 2019). Hence, the transcriptional responses of *Daphnia* to environmentally exposed NMs are a rich source of both phenotypic and genotypic information about the mechanisms of adaptation. This approach aligns human- and eco-toxicology towards a more general understanding of how exposure to NMs disrupts biological processes that otherwise ensure animal (including human) health. Evidence is growing on the feasibility of classifying the effect of NMs on humans, based on gene expression monitoring using distantly related environmentally relevant model organisms such as *Daphnia* (Amorim et al., 2023). Therefore, we can use comparative genomics to confirm that model organisms retain a greater number of ancestral gene families that are highly conserved and are shared with humans which are also closely linked to human diseases (Marwal and Gaur, 2020; Colbourne et al., 2022). Consequently, a chemical hazard assessment framework built upon key events may be informative for a greater diversity of species, by exploring the use of the homology between ecological model test species and humans to understand original molecular interactions and responses to emerging pollutants such as MPs and NMs.

### 5.2 Conserved biochemical pathways as a basis for understanding NM toxicity

The challenging ecological environments in which the multiple species of *Daphnia* inhabit, make *Daphnia*, an optimal genomic model for monitoring stress and adaptive changes (Vandegehuchte et al., 2010; Vandenbrouck et al., 2010; Colbourne et al., 2011) to their reproductive nature (as discussed in section 4). Understanding the genomic traits in *Daphnia* has already given great insight into developmental plasticity, causing altered morphology and behaviour in response to environmental stress (Akbar et al., 2022; Sha and Hansson, 2022) and adaptation to environmental toxicity (van Straalen and Feder, 2012). Therefore, due to their high degree of phenotypic plasticity, physiology and ecological importance (Lee et al., 2019), understanding the mechanism of action (MOA) as a result of daphnids exposure to NMs, is critical for the prediction of the selectivity and sensitivity in other species. Furthermore, this understanding will lead to the development of adverse outcome pathways (AOPs), by understanding what NMs concentrations cause harm (Russo et al., 2018), which can then be imposed onto standardized toxicological tests and risk assessments.

Access to the *Daphnia* genome sequence has enabled researchers to study specific gene changes in response to a multitude of environmental influences, and to discover the MOAs of several chemicals (Garcia-Reyero et al., 2009; Garcia-Reyero and Perkins, 2011; Giraudo et al., 2017) rendering gene transcription profiling one of the most powerful tools in developmental biology. The advancement of high-throughput RNA-sequencing (RNA-seq) provides whole transcriptome profiling, which allows the unbiased detection of novel transcripts in a sample at a given time (Giraudo et al., 2017).

Over the past decade, developmental work using phylogenetic mapping relationships and amino acid homology have identified that genomic regions under natural selection show evolutionary relationships among conserved genes from different species (van Straalen and Feder, 2012). Although there are major differences between vertebrates and invertebrates, there is a growing body of evidence that distantly related species share many ancestral genes by common descent that serve the same biochemical pathways (Rogozin et al., 2014). The homology between genes (similarity in genes due to shared ancestry between species) are analysed using Gene Ontology (http://geneontology.org/), gene orthologs database (https://www.orthodb.org/), or pathway enrichment tools (such as DAVID, GSEA or Reactome). This analysis catalogues genes shared among species by descent across the animal kingdom to infer functional conservation, which helps to highlight highly conserved key genes involved in stress response pathways. Therefore, the homology-based approach identifies genes “in common” from model species and aligns them with similar orthologues to gene family members across large evolutionary distances (Spanier et al., 2017; Adrian-Kalchhauser et al., 2020). Comparative genomics studies have confirmed that crustaceans retain a greater number of ancestral gene families that are shared with humans than insects (such as *Drosophila*), including genes responsible for growth, reproduction, and maintenance (Rogozin et al., 2014; Colbourne et al., 2022). Indeed, *Daphnia* retain a disproportionately large number of ancestral gene families that are linked to human diseases, despite more than 780 million years since present day crustaceans and mammals last shared a common ancestor (Colbourne et al., 2022).

Several studies have presented preliminary links, using qualitative gene expression profiling and *Daphnia*, between NM exposure-related harm and human disease (Asselman et al., 2013; Aksakal and Arslan, 2019; Ellis et al., 2020b). Such research uses gene expression to help bridge the gap between distantly related species by understanding how exposure to pollutants disrupts key conserved biological processes (Mav et al., 2018). The most common homologous genes observed in these studies have been those associated with metal detoxification, oxidative stress, energy production, DNA repair (Poynton et al., 2007) and general maintenance (Asselman et al., 2013; Qiu et al., 2015), which all served as biomarkers that are shared between species. The results from these studies collectively recognise that genes associated with growth, reproduction, homeostasis, xenobiotic detoxification, and metabolism, all provide mechanistic insights into NM-organism interactions and represent pathways encoding for cellular functions that are known to be induced by NM exposure studies.

Although further research is needed, comparing common genes and the biochemical pathways that link the differential transcriptomes, shared by common descent among species, offers meaningful insights into the connections between model test species and environmental health exposure, which can help to establish areas for development in NM risk assessment.

## 6 Adverse outcome pathways—gaps in terms of ecotoxicity and NMs

Relating molecular responses to phenotypic effects is crucial in environmental risk assessment. A promising approach is the AOP framework, which describes key steps of toxic mechanisms resulting in adverse effects in animals and populations (Ankley et al., 2010; Mortensen et al., 2022). AOP starts with a molecular initiating event (MIE) that can be triggered by various environmental contaminants. These MIEs are linked to a series of key events (KEs) that can result in an adverse outcome via different signalling pathways with different levels of biological organisation, e.g., molecular, cellular, tissue, organ, organism, and population (Villeneuve et al., 2014; Rugard et al., 2020). These interlinked pathways can be assembled into AOPs and serve as a foundation for the development of a mechanistic understanding of toxicity and disease. Therefore, AOPs play an essential role in the ecological risk assessment of environmental contaminants (Ankley et al., 2010; Khan et al., 2020).

To date, the majority of AOP-building effort has been focussed in human health and mammalian studies, but the application of AOPs for model organisms such as *Daphnia* is increasingly being investigated (Song et al., 2020). While in principle AOPs are chemical-agnostic, and focussed more on mechanisms, such as endocrine disruption or ion-channel blocking, for example, work is underway to establish NMs-specific AOPs, focussing on key aspects such as NMs-biomolecule interactions, NM-membrane interactions, and NMs-induced disruption of enzyme activity, for example, Jagiello et al. found that only 8 of 331 available AOPs in the AOP-Wiki are specific for NMs (namely, AOP numbers 144, 173, 207, 208, 209, 210, 241, and 319), meaning that only 2.4% of all AOPs in the AOP-Wiki have (as of 2022) been assessed or considered for their direct relevance to NMs, and thus there is a need to evaluate whether remaining AOPs might be applicable fully or partially to NMs (Jagiello et al., 2022).

NMs induced formation of ROS is one of the most significant reasons for adverse effects from NMs. Likewise, oxidative stress is well known to contribute to pollutant-induced cell damage and toxicity (Balážová et al., 2020). ROS can target multiple cellular components, including mitochondria, membrane lipids, DNA, structural proteins, and enzymes, resulting in different adverse outcome. NM exposure to *Daphnia* has caused NM accumulation in the gut, which led to accumulated ROS (Kim et al., 2010; Nasser et al., 2020), decreased growth (Karimi et al., 2018; Ellis et al., 2020b), and decreased fertility and reproduction (Mendonça et al., 2011; Olkova, 2022). When Cu (II) was absorbed on TiO_2_, the oxidative stress increased, and intestinal damage was found (Liu et al., 2015). It has been reported that CNT exposure can cause ingestion of CNTs, leading to impacted gut and poor food assimilation, which in turn, may lead to poor nutrition and cause adverse effects to growth, moulting, and eventually reproduction (Arndt et al., 2014). Thus, it is likely that gut accumulation of NMs can lead, via oxidative stress, reduced growth and thus reduced fertility, to reduced reproductive success and population decline. Within the RiskGONE, NanoSolveIT and CompSafeNano projects we are documenting the key events in this proposed AOP for discussion with the AOP community.

NMs accumulation in the gut of *Daphnia* can cause blockage of gut, leading to reduced feeding, followed by decreased supply of oxygen and triggering of antioxidant pathways (e.g., decreased SOD, upregulation of NOX5, increased ROS), which result in decreased moulting and thus the decreased growth, fertility, and reproduction (Sasaki et al., 2019). ROS production induced oocyte apoptosis-associated reproduction decline has been reported previously, whereby increased ROS damages cellular components, leading to energy (ATP) shortage, DNA breaks, telomere shortening, spindle instability, chromosomal abnormalities, dysregulation of autophagy and proteasome system, which may contribute to the reduced developmental competence compared to normal oocytes (Sasaki et al., 2019). In addition, excessive ROS formation can induce increased DNA damage, apoptosis, follicular atresia, and decreased oogenesis, reducing fecundity (Chatterjee and Bhattacharjee, 2016). Therefore, excessive ROS production can lead to oocyte apoptosis-associated reproduction decline. Calorific reduction related to NMs accumulation in the gut (as discussed in Section 4) may also trigger a cascade of signalling pathway alteration, including inhibition of FAR gene expression, activation of DMRT and DMRT genes and sex determination genes (doublesex1), and changes in chitin and ceramide metabolism, which subsequently lead to decreased carapace shedding, *Daphnia* maturation, sex communication, and induced male production.

We propose that all these KEs are triggered by the MIE of physical blockage of the gut. Absorption of NMs onto the surface of aquatic organism is a key step in determining their bioavailability. Absorption of AuNMs on the carapace and appendages of *D. magna* and the resulting mechanical disruption of the feeding appendages was observed after AuNM exposure (Botha et al., 2016). TiO_2_ NMs were taken up mainly by endocytosis, resulting in their accumulation in abdominal areas and the gut of *D. magna* (Tan et al., 2017). Elimination of NMs is reportedly difficult; the excretion rate constant of AgNMs in daphnids was much lower than that of Ag ions (Zhao and Wang, 2010). The main elimination routes for AgNMs in *Daphnia* were excretion (63%) and faecal production (Zhao and Wang, 2010). The monodispersed NMs can easily get deep inside the organisms and are harder to be excreted compared with aglomerated NMs.

The application of omics technologies can be of great value for elucidating how contaminants cause adverse effects in an organism. For example, proteomics provides a systematic qualitative and quantitative mapping of the whole proteome in cells and tissues and enables identification of differentially expressed proteins (DEPs) as biomarkers for AOPs. In addition, transcriptomics (single organism), metabolomics, and lipidomics can also be used for future studies for identifying the AOPs (Ankley et al., 2010; Vinken, 2019).

## 7 Innovative approaches to assessing NMs toxicity using *Daphnia* microfluidics

Microfluidics and lab-on-a-chip are promising technologies to address many of the limitations in toxicity assays. With these innovative approaches the manipulation of small volumes of liquids/fluids under a network of miniaturized channels allows them to 1) mimic the microenvironment conditions, 2) enables easy manipulation of cells and organisms to measure biological specimens and biomolecular targets, and 3) facilitates automatic extraction of relevant data in a fast and easy way. Additionally, these devices can be combined with sensors, cameras, computers, and smartphones becoming a powerful toolbox in toxicological sciences. Initially, lab-on-a-chip devices were applied in toxicity studies to miniaturize and refine *in vitro* assays. These devices reduce and automate manual handling procedures, miniaturize testing, decrease the required amount of reagent and chemicals for testing and improve performance due to the real-time monitoring capability. Microfluidics technology creates the potential for multiplexed analysis, single-cell, and gradient cytotoxicity assessments. Furthermore, this technology combines sensors and digital cameras for cytotoxicity studies with real-time data collection, allowing the monitoring of many cellular parameters, such as mortality, cell-substrate adhesion, electrophysiology, cell division and kinetics responses of cytotoxicity in a label-free manner (Garcia-Hernando et al., 2020; Wlodkowic and Jansen, 2022).

Despite the evolution of microfluidics devices and lab-on-a-chip technologies in the biomedical field, its potential in ecotoxicology has emerged just recently. Ecotoxicology testing using *in vivo* assays is, in general, labour intensive, whereby the manipulation of organisms is mainly manual, which may increase data variability and decrease reproducibility. In this way, microfluidics automation of organisms sorting, collection and positioning and chemical addition and dilution, combined with powerful data collection and analysis tools offer a significant upgrade to ecotoxicological tests (Campana and Wlodkowic, 2018a; Abreu et al., 2022). However, there are also some limitations for its development, such as organism size, which is an important parameter to the development of micro/millifluidic devices, currently limited to organisms < mm in size (Campana and Wlodkowic, 2018b). Consequently, a limited number of studies combine micro/millifluidics with ecotoxicology, and most address unicellular organisms such as bacteria, algae or protozoa. Such devices can detect growth, cell viability, bioluminescence, movement, and electrochemical changes to understand the mechanisms of toxicity for those model organisms (Kim J. et al., 2017; Zhang et al., 2017; Altintas et al., 2018). For multicellular organisms, some ecotoxicity studies were reported, for example, with: rotifers (*Brachionus calycifloru*s) (Cartlidge and Wlodkowic, 2018), Crustacea (*Artemia* sp.) (Huang et al., 2015)*, Allorchestes copressa* (Cartlidge et al., 2017) and *Daphnia magna* (Huang et al., 2017; Tabatabaei Anaraki et al., 2018)*,* nematode (*Caenorhabditis elegans*) (Kim J. et al., 2017; Zhang et al., 2021; Aubry et al., 2022) and fish (*Danio rerio*) models (Yang et al., 2016; Khalili and Rezai, 2019).

Traditional ecotoxicology assays evaluate the survival, reproduction, or growth rate at a specific time point (hours/days) and estimate the chemical median lethality (LC_50_) or effective concentrations (EC_50_). Exploring microfluidics technology, it is possible to refine the analysed toxicological parameters and assess different preliminary responses and/or obtain real-time mortality rates. For example, behavioural parameters are often more sensitive than physiological, developmental or reproductive endpoints (Melvin and Wilson, 2013). Therefore, microfluidics technology can increase data analysis and collection by caging test specimens in miniaturized devices allowing the observation of mobility and/or swimming alteration (Bai et al., 2018). Another limitation for classical assays is the caging and manipulation of organisms for imaging, or measurements that need the animal to stay still, such as optical imaging, size and heartbeat measurements. For example, for *C. elegans* model microfluidic devices allowed immobilization by the restriction in thin microfluidic channels (Chokshi et al., 2009; Kim J. et al., 2017). Microfabricated devices for precise and controllable rotation of organisms, for analysis of specific body parts, imaging or injection of substances, were already fabricated for *C. elegans* (Pan et al., 2021) and zebrafish models (Zhang et al., 2017).

**FIGURE 4:**
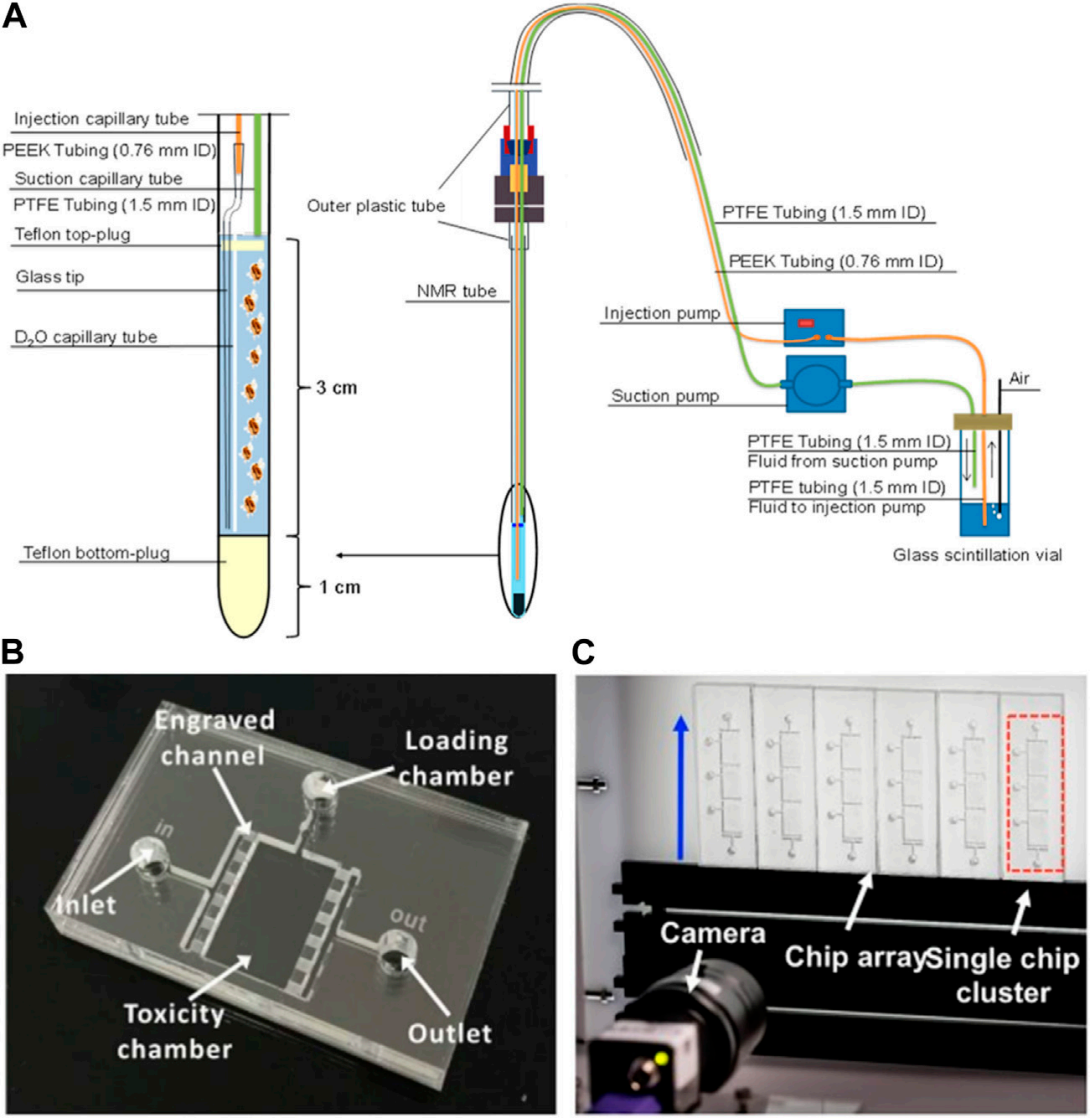
**(A)** Low-volume flow system for *D. magna in vivo* NMR (Tabatabaei Anaraki et al., 2018); **(B)** microfluidic chip for *D. magna* toxicity testing (Huang et al., 2015); **(C)** Photograph of the system setup consisting of a millifluidic chip-based array, fluid actuation and optical detection modules (Huang et al., 2017). Reproduced with permission from SCRIP and SPIE, respectively.

As for *Daphnia*, there is a complete open avenue for innovation using microfluidics and lab-on-chip technologies because there are just three reports in the literature (Figure 4). Tabatabaei Anaraki et al. (2018), developed a low-volume flow system that allows *D. magna in vivo* analysis under nuclear magnetic resonance (NMR) testing using a 5 mm NMR tube; inside the tube are two capillary tubes for injection and suction of fluids (sample, media and/or algae injection), allowing the exposure of the living organism to low volumes of chemicals for *in vivo* metabolomic studies (Tabatabaei Anaraki et al., 2018). Huang et al. (2015), automated the Daphtox kit-F with a microfluidic technology by developing a microchip, consisting of a toxicity chamber with a fluid inlet and outlet and loading chamber, for caging *D. magna.* The system was connected with a high-definition time resolved video data analysis to monitor *Daphnia* behaviour when exposed to CuCl_2_ as a model toxicant (Huang et al., 2015). The was further improved (Huang et al., 2017) to include an array of 24 cuboid test chambers, grouped in eight clusters of three chambers, each chamber having its own specimen loading port and interconnected chambers in a cluster have a shared inlet and outlet for media and sample injection. The device was connected with a time-resolved video microscopy and software to track and analyse *D. magna* locomotory responses towards pollutants (i.e., CuCl_2_, potassium dichromate, xanthine alkaloids (caffeine), ethanol and dimethyl sulfoxide) (Huang et al., 2017). Interestingly, these results showed that the milli-fluidic device (Daphtox II) presented an EC_50_ equivalent to the conventional multi-well plate acute toxicity assay but can assess multiple behavioural endpoints and provide a more sensitive test with higher automation than the conventional multi-well test.

Despite the enormous benefits that can be achieved by microfluidics applications in nanotoxicity assessment, there are still few studies addressing this subject. With the *Daphnia* model, to the best of our knowledge, there are only two toxicity studies with chemicals (Huang et al., 2015; Huang et al., 2017) and no reports involving NMs, daphnids and microfluidics at the moment. This knowledge gap has been initially addressed by Seitz et al. (2013) when studying the toxicity of TiO_2_ NMs towards *D. magna* (Seitz et al., 2013), who showed that in semi-static experiments the initial concentration of TiO_2_ decreased approximately 95% while in flow-through the concentration of TiO_2_ remained constant in the water column throughout the test duration, with a concurrent decrease in particle agglomeration and sedimentation, as determined by Dynamic Light Scattering. The flow-through conditions also showed lower toxicity of TiO_2_ NMs suggesting that agglomeration may play an important role in the toxicity profile of NMs (see Section 2.1). In addition, the NM sample is usually limited and expensive, and microfluidics devices can be useful not only in this sense (saving sample and producing less residues) but also in improving test conditions, increasing flow control, decreasing evaporation, controlling media oxygenation, temperature, etc. The fabrication of devices may require specific laboratory facilities and can be laborious, limiting mass production of devices for application in ecotoxicology currently (Campana and Wlodkowic, 2018a). However, this technology can obtain measurements and information that traditional assays are not able such as toxicity dynamics events, *in situ* analysis and real-time monitoring. Additionally, all the data generated can be automatically collected using specific software analysis and computational tools, supporting the implementation of big data and machine learning methods in nanoecotoxicology, especially, when considering *Daphnia* as a key organism model in nanosafety regulation.

## 8 Key recommendations and future directions

*Daphnia* have been well established as an important model organism for ecotoxicity testing due to their role in the ecosystem, rapid parthenogenetic reproduction, their responsiveness to xenobiotics and environmental stressors and the range of endpoints available to access for toxicity testing. In addition, their use in toxicity testing is compliant with the principles of reduction, replacement, and refinement (NC3Rs) of traditional animal testing makes them an ideal model organism to explore and develop methods for to evaluate emerging contaminants and concerns. Their historic use in chemical testing made them an ideal species to evaluate NM and MP toxicity in freshwater ecosystems, and as highlighted several advances have already been established in the field of NM and MP ecotoxicity assessments. The importance of characterisation of particles has been well established for NM, and lessons and best practice can be taken into MP research to further advance this field, including methods to quantify uptake and techniques to characterise particle surface which are have been identified as important aspects of ecotoxicity studies.

When evaluating the use of *Daphnia* as an ecotoxicology model to determine biological and environmental impacts of NMs and MPs, several developments in terms of both methodology and understanding emerged that offer enormous promise for the future. Highlights include the complete sequencing and elucidating the *Daphnia* genome which, when paired with the parthenogenetic reproductive pathways in *Daphnia,* enables detailed genetic responses to be determined and subsequent changes to offspring to be ascertained (Section 5). Although further research is required, the capacity to compare common genes and pathways across species, by comparing genes shared by common descent among species, and the biochemical pathways that link differential transcriptomes offers meaningful insights into the relationship between model species and environmental health exposure, which can help to identify areas for development in NM risk assessment and support the development of New Approach Methodologies and Next-Generation Risk assessment approaches that rely far less on mammalian *in vivo* testing (Ellis and Lynch, 2020). In addition to transcriptomics of single organisms, application of omics techniques can further support the development of AOPs for *Daphnia* populations and whole ecosystems in response to exposure to NM and MPs, the use of proteomics, metabolomics, lipidomics can help to link the responses to MIEs (Section 6).

Additionally, new methodologies such as microfluidic and lab-on-chip technologies have been identified as areas ripe for development in NMs ecotoxicology. The use of microfluidics can enable real time monitoring of physico-chemical properties of the NM in addition to the toxicity response of the organism. The potential for NM to agglomerate and sediment in test systems can be overcome by the optimised flow conditions that can be established in the microfluidic systems compared to the tradition static test system set up (Section 8). Furthermore, data collection during this application is automatic, enabling machine learning methods and big data computations approached to evaluate the changes and response in due course, utilising harmonised and curated datasets produced according to the FAIR data principles (Wilkinson et al., 2016)*.* While developments of *in silico* models for NMs toxicity to *Daphnia* have been limited to date, and focus primarily on predicting acute toxicity (Varsou et al., 2021), exciting progress in terms of models for assessing impacts from mixtures of different types of NMs (Zhang et al., 2022) and on use of machine learning from images of daphnids exposure to NMs and assessment of changes in tail length, lipid deposits and other phenotypical characteristics from paired multi-generational studies comparing continuous versus parent-only NMs exposure (Karatzas et al., 2020) suggest that computational modelling is a very promising direction for the future.

There are also key areas that have been highlighted where adaptation or further development of approaches and methodologies would be beneficial to strengthen the capacity to evaluate the biological and environmental effects of NMs and MPs. Firstly, through ongoing efforts to adapt test guideline and toxicity study designs to take into consideration the surface characterisation and changes of NM and MPs that result from the exposure medium/conditions and the local environment. This has been demonstrated to have significant impacts on the chemical and biological signalling of the particles that can influence the subsequent interaction. Furthermore, the testing of particles that have been ‘aged’ in the biological/environmental test medium can lead to substantial changes to the observed toxicity response in both acute and chronic exposure. A balance between comparable/reproducible results and environmentally realistic exposure scenarios would address these challenges going forwards (Section 2), and a strong focus on knowledge transfer for NMs to MPs researchers is essential in order to prevent re-invention of knowledge.

Whilst developing the complexity of the testing conditions for particles, increasing the scale of exposure timeframes can also lead to significant changes to the observed toxicity. Multigenerational assessments to date have highlighted that the offspring of the exposed parents most often have increased sensitivity (and therefore observed toxicity) compared to the initially exposed parent. This suggests that there could be significant detrimental impacts to natural populations based on the assumption that the toxicity response of all daphnids would be within the range observed in initial acute (48-h short term exposures) and even chronic (21-day reproductive exposures) test windows when not considering the impact of subsequent generations (Section 3). Application of machine learning approaches might enable assessment of the impact of different media compositions and thus different underlying *Daphnia* fitness conditions, as well as the role of additional stressors, such as competition for food or climate change.

As particle uptake does not follow the octanal-water partition coefficient (log Kow) principles, the qualification of uptake of NMs and MPs is an important aspect of ecotoxicity studies to determine an accurate dose-response relationship, and to enable Toxicokinetic-Toxicodynamic (TK-TD) modelling which can link life history traits observed as a result of exposure to changes in the population dynamics in the ecosystem. There are several methods currently available, including TEM imaging, ICP-MS quantification of metal and metal doped particles and the use of fluorescence for stained particles, however the method used depends on the material of the particle (Section 4). However, there are limitations to the use of fluorescence, such as the leaching of fluorescence dye or the impedance of these methods based on the NM properties which can cause interference with assay read-outs including autofluorescence. As a result, further work into accurate methods for determining the internalised concentration of particles would be beneficial to make this a valuable source of data for machine learning.

## Abbreviations

AOPs, adverse outcome pathways; CAT, catalase; CB, carbon black; CNT, carbon nanotube; DEB, Dynamic Energy Budgets; EPS, extracellular polymeric substances; FAIR, Findable, Accessible, Interoperable, Re-usable; GPx, glutathione peroxidase; GST, glutathione-s-transferase; KEs, key events; MDA, malondialdehyde; MIE, molecular initiating event; MOA, mechanism/mode of action; MP(s), microplastic(s); NM, nanomaterial(s); NOM, natural organic matter; QD, quantum dot; ROS, reactive oxygen species; SOD, Superoxide dismutase; TEM, Transmission electron microscopy; TK-TD, Toxicokinetic-Toxicodynamic.
